# The alternative splicing landscape of a coral reef fish during a marine heatwave

**DOI:** 10.1002/ece3.8738

**Published:** 2022-03-18

**Authors:** Stanley Kin Nok Chan, Sneha Suresh, Phillip Munday, Timothy Ravasi, Moisés A. Bernal, Celia Schunter

**Affiliations:** ^1^ Swire Institute of Marine Science School of Biological Sciences The University of Hong Kong Hong Kong SAR China; ^2^ 8001 Australian Research Council Centre of Excellence for Coral Reef Studies James Cook University Townsville Queensland Australia; ^3^ 8001 Marine Climate Change Unit Okinawa Institute of Science and Technology Graduate University Onna‐son Japan; ^4^ 1383 Department of Biological Sciences College of Science and Mathematics Auburn University Auburn Alabama USA

**Keywords:** cellular physiology, climate change, molecular acclimation, thermal stress, transcriptome

## Abstract

Alternative splicing is a molecular mechanism that enables a single gene to encode multiple transcripts and proteins by post‐transcriptional modification of pre‐RNA molecules. Changes in the splicing scheme of genes can lead to modifications of the transcriptome and the proteome. This mechanism can enable organisms to respond to environmental fluctuations. In this study, we investigated patterns of alternative splicing in the liver of the coral reef fish *Acanthochromis polyacanthus* in response to the 2016 marine heatwave on the Great Barrier Reef. The differentially spliced (DS; *n* = 40) genes during the onset of the heatwave (i.e., 29.49°C or +1°C from average) were related to essential cellular functions such as the MAPK signaling system, Ca(2+) binding, and homeostasis. With the persistence of the heatwave for a period of one month (February to March), 21 DS genes were detected, suggesting that acute warming during the onset of the heatwave is more influential on alternative splicing than the continued exposure to elevated temperatures. After the heatwave, the water temperature cooled to ~24.96°C, and fish showed differential splicing of genes related to cyto‐protection and post‐damage recovery (*n* = 26). Two‐thirds of the DS genes detected across the heatwave were also differentially expressed, revealing that the two molecular mechanisms act together in *A*. *polyacanthus* to cope with the acute thermal change. This study exemplifies how splicing patterns of a coral reef fish can be modified by marine heatwaves. Alternative splicing could therefore be a potential mechanism to adjust cellular physiological states under thermal stress and aid coral reef fishes in their response to more frequent acute thermal fluctuations in upcoming decades.

## Graphical abstract

A wild coral reef fish, *Acanthochromis polyacanthus*, exhibited different RNA‐splicing patterns in their livers across three major periods of a marine heatwave. The functions of differential spliced genes indicated that alternative splicing could be a potential mechanism to adjust cellular physiological states under thermal stress and aid coral reef fishes in their response to more frequent acute thermal fluctuations in the upcoming decades.

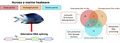

## 1 INTRODUCTION

Human‐induced global warming is a considerable challenge for marine organisms, as it is expected to push species beyond their physiological limits, causing irreversible changes to population and communities in many marine ecosystems (Hoegh‐Guldberg & Bruno, 2010). Alongside increasing average ocean temperatures, marine heatwaves are also increasing in intensity and frequency (Gupta et al., 2020; Oliver et al., 2018), and events have surged more than twentyfold since the 1970s due to anthropogenic climate change (Laufkötter et al., 2020). Marine heatwaves are an abnormal period of warming in the ocean, which can last days, weeks, or months, where temperatures exceed the normal seasonal range (Hobday et al., 2016). Even with a short duration, these extreme thermal events affect the physiology of individual organisms and can have cascading consequences on populations, communities, and the overall biodiversity of a specific area (Fordyce et al., 2019; Oliver et al., 2019; Smale et al., 2019). Given the serious impact marine heatwaves can have on poikilotherm organisms, it is fundamental to evaluate the physiological changes and the underlying molecular mechanisms that animals use to adjust to these extreme thermal events (Hofmann & Todgham, 2009).

One example is the recent heatwave that occurred in the austral summer of 2015/2016, which led to coral bleaching and subsequent loss of great expanses of coral reefs throughout the Great Barrier Reef in Australia (Hughes et al., 2018). The coral die‐off and subsequent changes in community structure as a result of the heatwave influenced the trophic dynamics of the ecosystem, ultimately reducing the diversity of coral reef associated organisms (Wilson et al., 2019). This warming event also affected the survival, development, reproduction, and feeding patterns of different coral reef fishes (Genin et al., 2020; Piatt et al., 2020; Spinks et al., 2019; Triki & Bshary, 2019), ultimately leading to a reduction in fish biomass along the Great Barrier Reef (Brown et al., 2021).

Marine organisms have various strategies to cope with thermal stress. In the case of fishes, warming can lead to an increase in metabolic activity that usually translates into an increase in oxygen consumption (Pörtner et al., 2017). The changes in aerobic demand can stimulate a wide variety of biological changes in fish, including changes in glucose supply, hematological parameters, ion balance, and immunological functions (Sopinka et al., 2016) that could assist them in coping with elevated temperatures. In this process, however, other essential functions such as immune response, growth, or reproduction may be compromised, as changes in energy allocation are required for compensation (Alfonso et al., 2020). Fish species that are not able to sustain the aerobic demand will experience a reduction of aerobic scope, which could eventually affect their survival (Nilsson et al., 2009, 2010; Pörtner & Farrell, 2008; Pörtner & Knust, 2007). Thus, marine heatwaves can lead to relevant physiological changes as fishes respond to thermal stress.

Transcriptional activity is the cellular base of how organisms respond to external stimuli. Studies indicate that transcriptional responses to heat stress in marine fish can vary depending on the magnitude and duration of exposure to high temperatures (Liu et al., 2017; Uren Webster et al., 2018). Furthermore, previous studies of gene expression have described changes in the transcriptional program following exposure to warming at different life stages and for different durations: acute marine heatwaves exposure in adults (i.e., days to weeks; Bernal et al., 2020), developmental exposure in juveniles (i.e., months; Veilleux et al., 2015; Bernal et al., 2018), and cross‐generational exposure in adults and juveniles (i.e., months for each generation; Shama et al., 2016). Some studies have alternatively revealed conserved cellular stress responses associated with protein turnover, metabolic shifts, and response to oxidative stress among multiple fish taxa (Iwama et al., 1998; Logan & Buckley, 2015). By analyzing transcriptomics across fish families (Komoroske et al., 2021; Logan & Buckley, 2015), changes in processes like metabolism, oxygen delivery, and response to reactive oxygen species may indicate signatures of acclimation to elevated temperature over multiple generations (Bernal et al., 2018; Veilleux et al., 2015). Hence, the analysis of underlying molecular mechanism associated with responses to warming can help elucidate the biological responses and acclimation potential of fish to marine heatwaves.

While understanding the molecular responses of fish to marine heatwaves is crucial, little attention has been given to RNA splicing. This fundamental molecular process consists of the selective removal of introns and/or exons from the pre‐mRNA resulting in the formation of a mature mRNA. An alternative splicing process can produce different mature‐mRNA isoforms, which can change the functions of the final protein by introducing new functional domains or altering the conventional protein structures (Kelemen et al., 2013) or change the stability of the mRNA hence changing the protein level (Smith et al., 1989). Alternatively, spliced RNAs containing a premature‐stop‐codon may be degraded via nonsense‐mediated decay, which results in the regulation of gene expression (Fursham & Makeyev, 2014). RNA splicing facilitates the production of multiple mRNA isoforms from a single gene thereby diversifying the proteome and promoting plasticity of the transcriptome to respond to changes in environmental conditions (Chaudhary et al., 2019; Mastrangelo et al., 2012). Despite a raising awareness of the role of RNA splicing plays in the responses to environmental fluctuations (Healy & Schulte, 2019; Li et al., 2020; Tan et al., 2018; Wan & Su, 2015; Xia et al., 2018), its role in the context of thermal fluctuation remains unclear in marine fishes due to the low number of studies that have focused on these relevant taxa.

In this study we re‐analyzed the transcriptomic data from Bernal et al. (2020) to evaluate patterns of alternative splicing and differential gene expression of a coral reef fish across four time‐points during the 2015/2016 marine heatwave on the Great Barrier Reef (GBR), Australia. The study focused on the spiny chromis, *Acanthochromis polyacanthus* (Pomacentridae; Bleeker, 1885), which is a common planktivorous fish on the GBR and coral reefs in the Indo‐Australian archipelago (Randall et al., 1997; Thresher, 1985). We examined splicing patterns in the liver of *A*. *polyacanthus* across three main periods of the heatwave: (1) onset of the heatwave with the initial temperature increase (“onset period”; Dec to Feb), (2) period with a prolonged elevated temperature during the heatwave (“prolonged period”; Feb to Mar), and (3) period with temperature decline after the heatwave (“decline period”; Mar to Jul). This study focused on the transcriptional program of the liver, as this tissue is known to correlate well with the aerobic demands observed for fish exposed to warmer conditions (Smith et al., 2013). This study aims to identify the alternative splicing pattern of *A*. *polyacanthus* to a marine heatwave, potentially revealing one of the mechanisms used by fishes to compensate for temperature increase in coming decades.

## 2 METHODS

### 2.1 Fish collection, tissue extraction and RNA‐sequencing

Individuals of *A*. *polyacanthus* with a mean standard length of 57.3 ± 12.92 mm were collected by scuba diving at Palfrey Island, near Lizard Island in the northern Great Barrier Reef (GBR), Australia (14°41′39.1″S, 145°27′05.3″E) as previously reported by Bernal et al. (2020). Briefly, five individuals were collected at the same site at each of the four different time points across day time hours: before the heatwave (8^th^ to 10^th^ December 2015; monthly temp. = 28.4 ± 0.75°C), the beginning of the heatwave (20^th^ to 22^nd^ February 2016; monthly temp. = 29.5 ± 0.51°C), during the extended period of warming (18^th^ to 20^th^ March 2016; monthly temp. = 29.7 ± 0.53°C), and during the Austral winter (18^th^ to 20^th^ July 2016; 25.0 ± 0.24°C). The intervals for these four timepoints covered the main three periods (onset, prolonged, and decline period) of the heatwave. Fish were collected using clove oil anesthetic, euthanized, and transferred to the boat (James Cook University Animal Ethics approval A2408) where liver tissues were dissected, snap‐frozen in liquid nitrogen, and permanently stored at −80°C back in the laboratory until further processing. *A*. *polyacanthus* was selected due to its high relative abundance in the fish community composition in the GBR, the available information from studies in captivity (Bernal et al., 2018; Donelson et al., 2016; Veilleux et al., 2015) and being found to be sensitive to warming during the marine heatwave event (Bernal et al., 2020). To determine the approximate temperature range experienced by the fish during the heatwave, records from 2015 to 2016 were obtained from a sensor in Lizard Island (0.6 m of depth) operated by the Australian Institute of Marine Science (http://weather.aims.gov.au/#/station/1166; Data S1).

Total RNA was extracted by homogenizing the whole liver for one minute in a Fisherbrand Bead Beater with single‐use silicon beads and using up to 30 mg in RNeasy Mini Kits (Qiagen). DNA contamination was removed by on‐column digestion using DNAse I (Qiagen) following the manufacturer's instructions. RNA quality was evaluated with an Agilent Bioanalyzer and only samples with a minimum RNA integrity number of 6.5 and 1ug of RNA were further processed. Paired‐end fragments of 150 base pairs were sequenced with an Illumina HiSeq4000 at Macrogen, South Korea. These samples were sequenced with other species of coral reef fish described in the manuscript by Bernal et al. (2020). Individuals collected at different time points were randomized and sequenced in different Illumina Lanes (no more than two individuals per collection point per lane) to avoid the sequencing biases.

### 2.2 Sequencing and splicing analysis

The raw RNA‐Seq reads were assessed for quality with FastQC v0.11.9 (Andrews, 2010), and low quality reads and adapters were trimmed with Trimmomatic v0.39 (Bolger et al., 2014) by using the following parameters: “ILLUMINACLIP: TruSeq3‐PE‐2.fa:2:30:10:8:true LEADING:4 TRAILING:3 SLIDINGWINDOW:4:15 MINLEN:40”. Potential fungal, bacterial, and viral contamination was removed with Kraken v2.1.0 (Wood et al., 2019) using the standard database and a confidence score of 0.3. The high‐quality and clean sequences were then mapped to the *A*. *polyacanthus* reference genome (NCBI BioProject PRJNA690095) using STAR v2.7.6a (Dobin et al., 2013) in the 2‐pass mode using default parameters.

**FIGURE 1:**
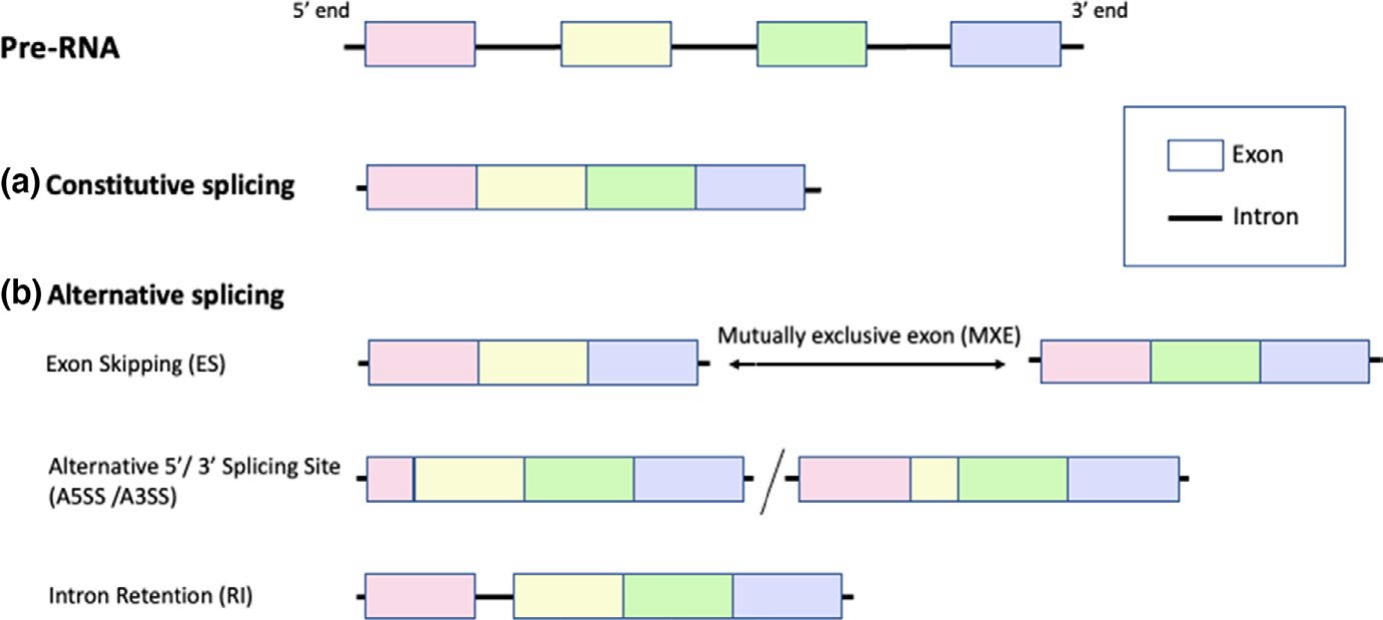
Illustration of the splicing mechanism of pre‐RNA. As a post‐transcriptional modification mechanism, a pre‐RNA molecule can be spliced into mature‐RNA by (a) constitutive splicing, or (b) five main ways of alternative splicing: Exon skipping (ES) or simple mutually exclusive exons (MXE); alternative 3’ splicing site (A3SS), alternative 3’ splicing site (A5SS) or intron retention (IR)

To evaluate the general splicing landscape of *A*. *polyacanthus*, the program AStalavista (Foissac & Sammeth, 2007) was run using default settings. The five most prevalent forms of alternative splicing were evaluated: exon skipping (ES), intron retention (IR), alternative 5′ splice site (A5SS), alternative 3′ splice site (A3SS), and mutually exclusive exons (MXE; Figure 1). As there are many forms of MXE that can involve multiple exons, we only consider the simplest form of MXE that only involves two adjacent exons, with the code “1–2^,3–4^” defaulted by AStalavista, while other complex forms of alternative splicing (including some of the rare MXE) were grouped into the “others” category.

Differential splicing (DS) events were then identified with rMATS‐turbo v4.0.1 (Shen et al., 2014), using both junction reads and reads mapping to exons. The analysis was conducted following pairwise comparisons of samples from different collection months: December versus February, February versus March, and March versus July. Exon inclusion levels (ψ), known as Percent Spliced‐In (PSI), of each exon was calculated as the relative abundance of isoforms which contains the target exon over the relative abundance of all isoforms. A likelihood‐ratio test was conducted, and genes were only considered as significantly differentially spliced when the adjusted *p*‐value with the Benjamini–Hochberg correction was less than 0.05 and the difference of the mean *ψ* of each gene between groups was greater than 0.1 under the three comparisons.

A gene ontology (GO) enrichment analysis was done for differentially spliced genes using a Fisher's exact test in Omicsbox with default settings (Conesa et al., 2005). Due to the small number of DS genes identified and incomplete annotation of the reference genome, the Gene Ontology (GO) enrichment analysis revealed no significant enrichment after multiple testing corrections after multiple testing correction (FDR < 0.05). Therefore, the functions of genes with putative splicing events were classified into functional categories based on the description in NCBI’s reference sequence (RefSeq) database (http://www.ncbi.nlm.nih.gov/RefSeq/), the UniProt KnowledgeBase (UniProtKB; https://www.uniprot.org/), and PANTHER16.0 (Mi et al., 2021); using the genome of *Danio rerio* as reference. As there were 29 orthologs not successfully annotated with the *Danio rerio* genome, they were subsequently mapped to *Mus musculus* reference genome. Categories were constructed by filtering out the keywords of the differential spliced genes appearing repeatedly in the descriptions in references belonging to biological systems (e.g., “Immune & inflammation system”) or functions (e.g., “Splicing”). The resulting categories allowed a wider and more comprehensive classification for describing the molecular functions involved in the response to warming. A heatmap was plotted to visualize the number of DS genes being categorized between groups of comparisons. The groups of comparisons were then clustered based on the pairwise Euclidean distance of the number of categorized genes across the categories. The process was done with the heatmap.2 function in gplots (v3.1.1; Warnes et al., 2020) in R v4.0.3.

For a more comprehensive understanding of molecular processes influenced by the temperature changes during the heatwave, differential gene expression analysis was performed to correlate with the differential splicing patterns. Gene expression was quantified by featureCounts (Liao et al., 2014) and imported into 3D RNA‐seq (Guo et al., 2020). For this, the low expressed transcripts were filtered (Count Per Million reads ≤ 1), the batch effects were reduced using the RUVr method, the data was normalized with weighted trimmed mean of M‐values, and the differential expression analysis was performed with Limma‐voom. This method models the mean‐variance relationship of the normalized read‐counts under an empirical Bayes approach (Law et al., 2014). This is a different approach than the one used for the analysis of differential expression in our previous paper (Bernal et al., 2020), as the current estimates with Limma adopt a more stringent normalization method for the DE genes. This will help us reduce the chances of false positives when linking the differential spliced genes to differential expressed genes. Limma has also shown better performance when comparing DE with alternative splicing, than simple count‐based RNA‐seq methods (Law et al., 2014). After these estimates, the log2 fold change (L2FC) of gene abundance was calculated based on contrast groups and significance of expression changes was determined using *t*‐test. *p*‐values of multiple testing were adjusted with Benjamini‐Hochberg procedure to correct the false discovery rate (FDR). A gene was significantly differentially expressed in a monthly comparison if it had adjusted *p*‐value < .05 and |L2FC| ≥ 1, as a default cutoff in 3D RNA‐seq.

## 3 RESULTS

### 3.1 Sequencing statistics

Mean values of 32.4 million (± 4.6 million) raw paired‐end reads was obtained across 20 samples with a mean Phred quality score above 30. On average, 0.6% of reads (0.2 million ± 0.1 million) were identified as contamination with Kraken and were removed. The remaining, 32.3 million (±4.6 millions) reads on average per individual were used for mapping and identification of AS and DS events. On average, 28.2 million reads (87.3%) were uniquely mapped to a single genomic region, and 1.6 million reads (5.0%) were multi‐mapped (Data S2).

### 3.2 The alternative splicing landscape of *A. polyacanthus*

**FIGURE 2:**
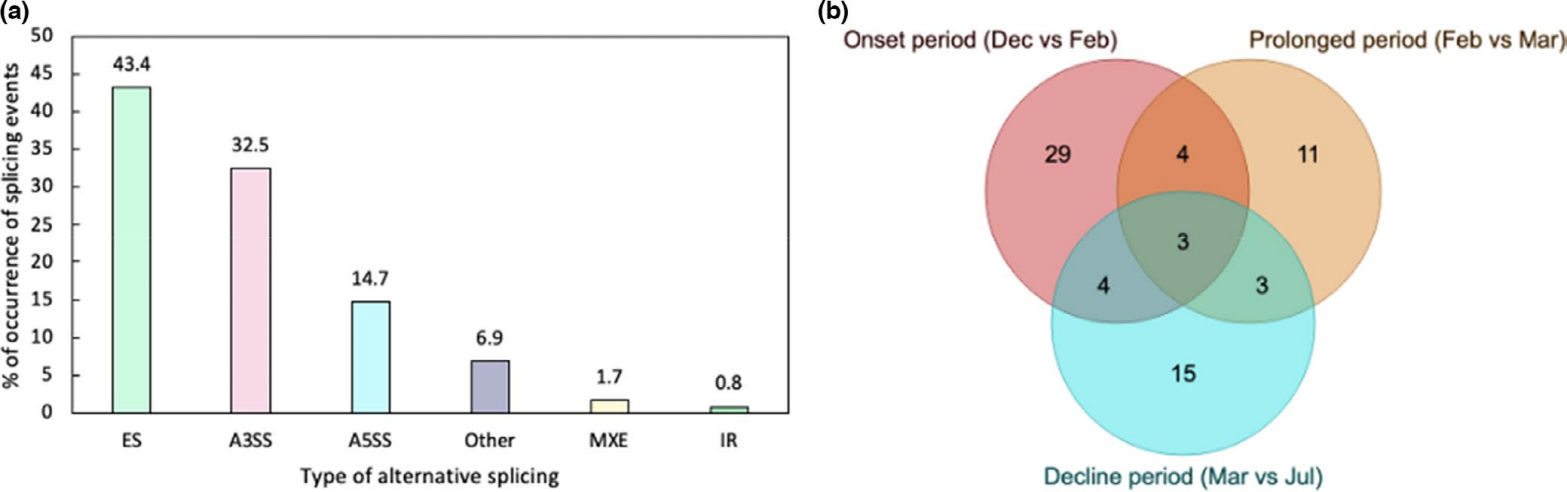
(a) Splicing landscape for all samples of *Acanthochromis polyacanthus*. The percent of occurrence of each of the categories: Exon skipping (ES), alternative 3’ splicing site (A3SS), alternative 3’ splicing site (A5SS), intron retention (IR), simple mutually exclusive exons (MXE), and other complex types are presented. (b) Venn diagram showing the number of differentially spliced genes per time‐point across the heatwave and the overlap in differentially spliced genes between the comparisons

Among the 34,194 annotated genes in the *A*. *polyacanthus* genome, a total of 15,402 genes were found to be expressed in our samples. Of the 15,402 genes, 1,980 genes (12.9%) were found to have more than one transcript (i.e., 16,593 transcripts in total). Among the splicing events estimated by AStalavista, exon skipping (ES) was the most abundant (990 events, 43.4%), succeeded by alternative 3′ splice site (A3SS; 741 events, 32.5%), alternative 5′ splice site (336 events, A5SS; 14.7%), other complex types (157 events, 8.3%), mutually exclusive exons (MXE; 39 events, 1.7%), and the least abundant was intron retention (IR; 18 events, 0.8%; Figure 2).

### 3.3 Differential splicing in response to the heatwave

In total, we identified 69 genes to be differentially spliced (DS) among all the three pairwise comparisons across the heatwave (Data S3). Among these identified DS genes, ES was the most abundant splicing type (42 genes), followed by MXE (17 genes), A3SS (10 genes), and A5SS (4 genes). No intron retention events were found across all pairwise comparisons (Table 1). Only one gene, *hemopexin* (*hpx*) was identified to have two splicing types (SE and MXE), while *15*‐*Hydroxyprostaglandin Dehydrogenase* (*hpgd*) was the only gene with three splicing types (A5SS, ES and MXE).

**TABLE 1:** A number of genes that were differentially spliced and their percentage, based on the types of differential splicing (DS) among the three collection periods of the heatwave (Data S4). Note that a DS gene could have more than one splicing type and occur in more than one period (% = percentage of DS genes occurred in that period over total number of gene among three periods for one DS type). Exon skipping (ES), alternative 3’ splicing site (A3SS), alternative 5’ splicing site (A5SS), intron retention (IR), simple mutually exclusive exons (MXE)

DS Type	Onset (Dec–Feb)		Prolonged (Feb–Mar)		Decline (Mar–July)		Total
	No. of genes	%	No. of genes	%	No. of genes	%	No. of genes
A3SS	6	60	2	20	2	20	10
A5SS	3	75	0	0	2	50	4
MXE	4	23.5	5	29.4	10	58.8	17
ES	27	64.3	14	33.3	15	35.7	42
IR	0	0	0	0	0	0	0
Total	40		21		25		69

A total of 40 genes were identified to be DS during the onset period of the heatwave (Dec vs. Feb), 21 genes were DS during the prolonged period (Feb vs. March), and 25 genes were DS during the decline period (Mar vs. July). Of these, 29 DS genes were unique to the onset period, 11 were unique to the prolonged period and 15 were unique to the decline period (Figure 2b). Four genes were commonly DS between the onset and prolonged stage: *CDC Like Kinase 4* (*clk4*), *Microtubule Affinity Regulating Kinase 3* (*mark3*), *Putative Monooxygenase* (*p33monox*), and *WAP Four*‐*Disulfide Core Domain 3* (*wfdc3*). Three genes were commonly DS between the prolonged stage and recovery stage: *Solute Carrier Family 31 Member 2* (*slc31a2*), *Collagen type I alpha 2* (*col1a2*), and *MAX Dimerization Protein* (*mga*). Meanwhile, four genes were commonly DS between the onset stage and recovery stage: *Heterogeneous Nuclear Ribonucleoprotein L* (*hnrnpl*), *Nuclear Transcription Factor Y Subunit Beta* (*nfyb*), *Signal Peptide Peptidase Like 2A* (*sppl2A*), and *TNFAIP3 Interacting Protein 1* (*tnip1*). A total of three genes including *Hemopexin* (*hpx*), *Inter*‐*Alpha*‐*Trypsin Inhibitor Heavy Chain 3* (*itih3*), and *Ribosome Binding Protein 1* (*rrbp1*) were alternative spliced across all the stages (Figure 2b).

**FIGURE 3:**
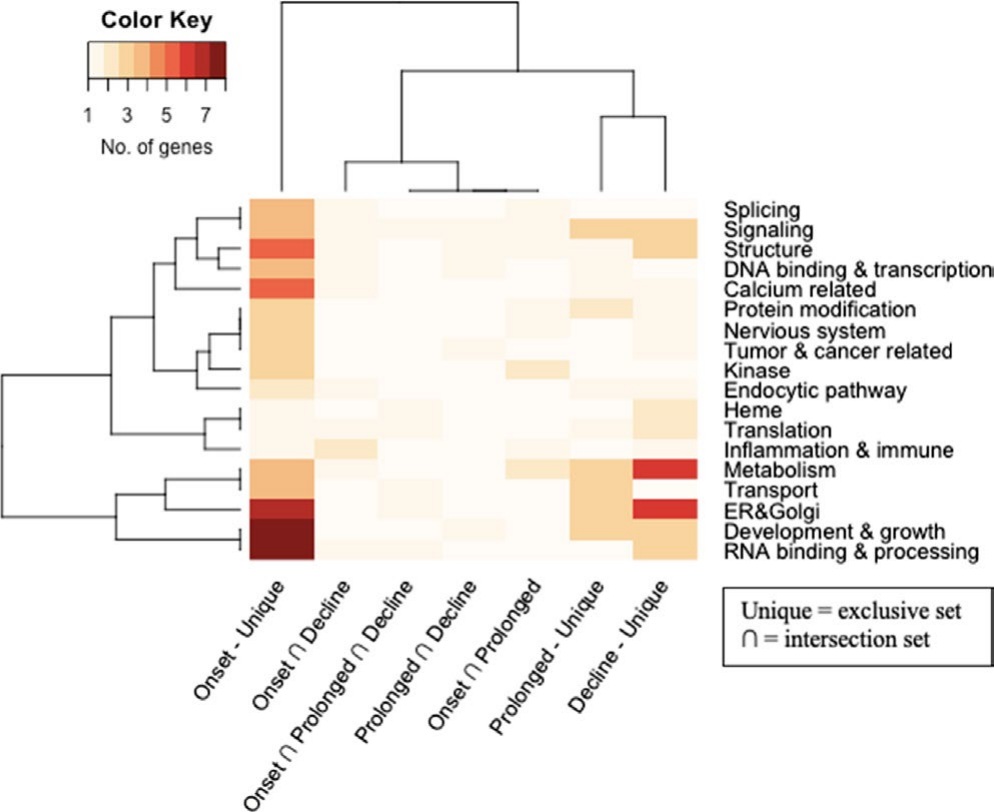
Heatmap representing the amount of differential spliced genes for each of the 18 functional categories associated with heat stress across different time‐point comparison, in which the comparisons were clustered based on the pairwise Euclidean distance of the number of categorized genes across the categories (Onset = onset of heatwave – Dec vs. Feb; Prolonged = prolonged period of heatwave – Feb vs. Mar; Decline = decline period of heatwave – Mar vs. July). (Unique = DS genes occurred only in a single period; ∩ = DS genes commonly occurred in different period)

The 69 significant differentially spliced (DS) genes were classified into 18 mutually inclusive functional categories (Data S5; Figure 3). The top five categories based on the number of DS genes were “Endoplasmic reticulum & Golgi apparatus” (16 genes), “metabolism” (16 genes), “development & growth” (15 genes), “signaling” (14 genes), and “RNA binding & processing” (13 genes).

### 3.4 Onset of the heatwave

In total, 40 genes were identified to be differentially spliced during the onset of the heatwave which was characterized by a sharp temperature rise in the average monthly temperature of 28.4°C to 29.5°C (Data S1). The DS genes were associated with defense mechanisms (i.e., apoptosis, immune and inflammatory response, and tumor suppression) including *mitogen*‐*activated protein kinase 14* (*mapk14a*), *structure specific recognition protein 1* (ssrp*1*), and *DAB adaptor protein 2* (dab*2*). Differentially spliced genes closely related to the endoplasmic reticulum and Golgi apparatus with endocytic pathway were also identified in the same period, such as *sec31 homolog A*, as well as genes responsible for Ca(2+) influx and maintenance of cellular structure such as *aspartate beta*‐*hydroxylase* (asph), *ras*‐*related protein 6A* (*rab6a*), *dystonin* (*dst*), *myosin IXB* (myo*9b*), and *microtubule actin crosslinking Factor 1* (*macf1*). We observed that there were multiple DS genes related to the mitogen‐activated protein kinase (MAPK) signaling pathway in the initial stage of the heatwave, including *mitogen*‐*activated protein kinase 14* (*mapk14*), *ER membrane protein complex subunit 10* (*emc10*), *TNFAIP3 interacting protein 1* (*tnip1*), *microtubule affinity regulating kinase 3* (*mark3*), and *complement component 3* (*c3*). It is noteworthy that the genes encoding for splicing proteins were themselves differentially spliced, including *KH*‐*type splicing regulatory protein* (*ksrp*; associated with a splicing enhancer), *survival of motor neuron 1* (*smn1*; plays a catalytic role in assembling the spliceosomal complex), *serine*/*arginine*‐*rich splicing factor 7* (*srsf7*; interacts with other splicing factors), and *CDC like kinase 4* (*clk4*; interacts with the serine/arginine‐rich splicing proteins).

### 3.5 Prolonged elevated temperature exposure

In the prolonged period between the collection points of February and March (i.e., exposure to warm temperatures for about a month), a total of 21 genes were significantly DS out of which 11 genes were unique to this period (Data S3). Some of these genes were associated with protein modifications: *Acid Phosphatase 1* (*acp1*), *Hexosaminidase Subunit Beta* (*hexb*), and *Microtubule Affinity Regulating Kinase 3* (*mark3*). Meanwhile, others were associated with metabolism: *Phenazine biosynthesis*‐*like domain*‐*containing protein 1* (*pbld1*) involved in isomerase activity, *LIM Domain 7* (*lmo7*) participates in ubiquitin‐protein transferase activity, and *Putative Monooxygenase* (*p33monox*) acts as potential NADPH‐dependent oxidoreductase.

### 3.6 Onset of winter after the heatwave

Twenty‐five DS genes were found (Data S3) during this period which had the largest temperature decline (4.74°C). These genes were associated with various functions, such as immune and inflammatory response: *complement component 3* (*c3*), *TNFAIP3 Interacting Protein 1* (*tnip1*), and *signal peptide peptidase like 2A* (*sppl2a*). Some were heme‐related: *5'*‐*Aminolevulinate synthase 2* (*alas2*) and *hemopexin* (*hpx*), which is involved in heme biogenesis and delivery, and c*ytochrome P450 2J5* (*cyp2j5*) and c*ytochrome P450 3A40* (*cyp3A40*), which are the cytochrome P450 genes binding to heme as oxidoreductase. Others were linked to DNA binding and transcription including *Ribosomal Protein S21* (*rps21*) and *ribosomal protein l36a* (*rpl36a*).

### 3.7 Differential gene expression associated with splicing patterns

A total of 9,189 genes were identified as differentially expressed (DE) across the heatwave periods, including 4,407 DE genes in the onset period, 4,186 DE genes in the prolonged period, and 5,238 DE genes in the decline period of the heatwave, respectively. Even when the main aim of this re‐analysis is to compare the DE in the context of DS, the DE analysis here reveals very similar results to the previous study on gene expression in *A*. *polyacanthus* (Bernal et al., 2020). Among the 69 DS genes, 46 DS genes (66.7%) were also found to be differentially expressed (DE) at least in one of the heatwave periods (Data S6). Specifically, 31 DS genes were DE in at least one of the heatwave periods that were here studied: 19 genes in the onset period, nine in the prolonged warming period, and 10 in the decline period (Data S7). This suggests that some genes involved in RNA processing and metabolism were regulated by both differential transcription and alternative splicing during the heatwave. For the top five categories of the DS genes, nine out of 16 DS genes were DE for “Endoplasmic reticulum & Golgi apparatus,” 10 out of 16 genes for “metabolism,” six out of 15 genes for “development & growth,” seven out of 14 genes for “signaling,” and 11 out of 13 genes for “RNA binding & processing.” This suggests that some genes involved in the categories were regulated by both differential expression and differential splicing but with varying intensity throughout the heatwave.

### 3.8 Exon splicing pattern of spliced genes over multiple periods

The exon splicing pattern of the DS genes varied among the different time points (Data S8). For the comparison between the onset and prolonged period, as well as between the prolonged and decline period, only a pair of isoforms was involved in each DS gene. These isoforms were spliced by exon skipping (ES) or mutually exclusive exons (MXE). The change of their inclusion level was moderate, except for *mga* which increased 0.754 in the onset period and decreased 0.547 in the prolonged period. Over all the periods, the inclusion level changes of *p33monox*, *mark3*, *mga*, *tnip1*, and *rrbp1* had an opposite trend to the expression level the in the onset period.

For the comparison between the onset and decline period, as well between all three periods, only the exons in *rrbp1* and *tnip1* were consistent, whereas other genes had more than one exon being spliced (i.e., more than a main pair of isoforms was involved). These exons were only spliced by the same mechanism applied on those spliced genes, except *hpx* of which three exons were spliced by ES and one was spliced by MXE. In addition, *hpx* demonstrated the most complex splicing pattern, where the exons were spliced in the onset and prolonged period, the onset and declined period, and only the decline period. The corresponding inclusion levels were also highly varied from 0.442 to −0.728 and, therefore, did not show clear correlation with the expression changes of *hpx* in all three periods.

## 4 DISCUSSION

Alternative splicing (AS) allows organisms to produce multiple transcripts from a single gene, representing an important mechanism for responding to external stimuli (Kwon et al., 2014; Laloum et al., 2018). For a more comprehensive understanding of the molecular responses associated with ocean warming, our study evaluated splicing patterns in the spiny chromis damselfish, *A*. *polyacanthus*, in response to the 2016 marine heatwave at the Great Barrier Reef. The results indicate that *A*. *polyacanthus* can implement both differential expression and AS to modify their molecular responses in response to a temperature change.

Without taking the collection points into account, the general splicing landscape observed in our non‐model species *A*. *polyacanthus* revealed a lower AS frequency than other teleost fish, such as zebrafish, medaka, fugu, stickleback, and catfish (Lu et al., 2010; Tan et al., 2019). While methods varied across studies, it is notable that the percentage of alternative spliced genes in fish genomes is highly variable: from 12.9% in *A*. *polyacanthus* (this study) and 17.0% in zebrafish to 43.2% in Fugu (Lu et al., 2010). A correlation between genome size and AS frequency has previously been suggested (Lu et al., 2010); however, *A*. *polyacanthus* has an intermediate genome size (1.0 Gb) in comparison to zebrafish (1.7 Gb) and Fugu (0.4 Gb) but reveals less AS. Considering the lower percentage of AS in *A*. *polyacanthus*, it is possible that the species relies less on alternative splicing to increase the protein diversity than other species. Still, one observation that concurs with alternative splicing among distant vertebrates (e.g., human, rat, and chicken; Kim et al., 2007) is that exon skipping (ES) is the most prevalent among the basic alternative splicing types in *A*. *polyacanthus*.

We found the largest number of DS genes between December and February, which represents the initial stage of the heatwave characterized by a sudden rise in temperature. These genes correspond to categories associated with protein synthesis and transport, as well as the interaction between the Endoplasmic Reticulum (ER) and Golgi apparatus. Thermal stress and the accompanying increase in aerobic demand in *A*. *polyacanthus* (Donelson & Munday, 2012) have been shown to result in the accumulation of reactive oxygen species (ROS) that can cause oxidative damage (Suzuki & Mittler, 2006). Oxidative stress can also disturb the functioning of ER, which may influence protein folding capacity and trafficking of intracellular proteins and can eventually lead to cell death. Evidence of this was seen with the gene *sec31 homolog a* (*sec31a*), which is responsible for formation of transport vesicles from the endoplasmic reticulum (Salama et al., 1997). This specific gene was differentially spliced during the onset period and was significantly down‐regulated in the onset period but up‐regulated in the prolonged period of the heatwave. Although a *sec31* homolog was previously found to be differentially spliced in response to heat stress in plants (Deng et al., 2016; Jegadeesan et al., 2018; Zhao et al., 2018), this is the first evidence for alternative splicing of this gene in fish under heat stress. Other DS gene, *RAB6A GTPase*, which regulate the intracellular vesicular trafficking from the Golgi apparatus to ER (Del Nery et al., 2006), was down‐regulated in the onset of the heatwave but up‐regulated in the prolonged period and decline period of the heatwave. These same patterns in the Golgi apparatus and ER were also detected in the original study that analyzed the gene expression dataset across the heatwave (Bernal et al., 2020), as well as the experiments in captivity with the same species (Bernal et al., 2018; Veilleux et al., 2015). Based on these results, we suggest that molecular regulation during the onset of the heatwave is associated with changes in the functions of the ER and Golgi apparatus, involving both differential expression and differential splicing.

Acute warming can lead to increased uptake of Ca(2+) by the mitochondria leading to Ca(2+) overload (Belhadj Slimen et al., 2014). This can lead to an increase in the reactive oxygen species (ROS) and reinforce the oxidative stress, forming a feedback loop leading to cell damage and death (Peng & Jou, 2010). Warming has also been shown to lead to the generation of lipid peroxides in a variety of fish species (Heise et al., 2006; Madeira et al., 2013; Vinagre et al., 2012). Lipid peroxidation is known for disrupting the cytoskeleton and damaging the mitochondrial membrane, causing cytotoxicity and apoptosis when mitochondrial calcium is released (Belhadj Slimen et al., 2014; Gardiner et al., 2013; Loven, 1988). Hence, differential splicing in genes related to Ca(2+) binding and cytoskeleton state in the onset period of the heatwave may play a role in cytoskeleton remodeling and restoring Ca(+2) homeostasis to counteract the consequences of heat stress.

A set of DS genes during the onset of the heatwave are associated with the Mitogen‑Activated Protein Kinase (MAPK) cascade. This signaling pathway is responsible for processes such as cell proliferation, development, and apoptosis (Guo et al., 2020). *Mitogen*‐*Activated protein kinase 14* (*mapl14*), *ER membrane protein complex subunit 10* (*emc10*), and *TNFAIP3 interacting protein 1* (*tnip1*) were three identified DS genes, whose expression was down‐regulated in both onset and prolonged period but up‐regulated in the decline period of the heatwave. These genes are related to p38 MAPK pathway which has a crucial role in the regulation of immune and inflammation response, as well as cell cycle and cytoskeleton remodeling (Cuenda & Rousseau, 2007). The transcriptomic changes may suggest that the fish experience activation of immune‐related genes after a drop in temperature after warming. Also, p38 MAPK has an alternative role in the indirect regulation of the activity of a differentially spliced gene *complement component 3* (*c3*; Maranto et al., 2008), which is associated with the innate immune system of bony fishes (Cheng et al., 2017; Demers & Bayne, 2020). As p38 MAPK could be responsive to heat stress (Nebreda & Porras, 2000; Whitmarsh, 2010), our results suggest the possibility that this signaling pathway related to immune response could be playing a role in the response to heat stress via alternative splicing and changes in expression.

The prolonged heatwave period with elevated temperatures over one month only yielded 11 uniquely differentially spliced genes, which is the lowest number among the heatwave periods. Genes that were uniquely differentially spliced in the prolonged period were associated with metabolism. It is possible that these metabolic changes to prolonged warming may help sustain the metabolic compensation resulted from an acute response of the onset period. However, there are few DS genes, and in contrast, over 4,000 genes were differentially expressed in the prolonged period (February vs. March), including genes associated with fatty acid biosynthesis, metabolic process, RNA processing, and respiration‐related mechanism (Bernal et al., 2020). This observation indicates that changes in splicing may be more relevant when individuals experience large changes in temperature over short time scales (days to weeks), as opposed to prolonged warming conditions. Yet, changes in gene expression may still play a much stronger role to compensate the effects of warming, as indicated by the large number of DE genes when elevated temperature conditions are maintained over longer periods of time (weeks to months). These results reveal that more studies are needed to understand which conditions trigger compensation via alternative splicing and which ones promote differential gene expression in marine ectotherms.

For the decline period of the heatwave a moderate number (26) of DS genes were found, which can be seen as a “recovery” period where fish transitioned from summer heatwave temperatures to winter with a decrease of 4.7°C. We identified DS in the genes *5'*‐*aminolevulinate Synthase 2* (*alas2*) and *hemopexin* (*hpx*). The former is related to heme biogenesis (Bailey et al., 2020) offering protection against oxidative stress (Liu et al., 2020), while the latter aids in delivering heme from the plasma to the liver for decomposition and iron recovery (Smith & McCulloh, 2015). Both hemoglobin and heme are central to the response to warming, as in *A*. *polyacanthus* an increase of approximately 1°C can lead to a significant increase in aerobic demand (Donelson & Munday, 2012; Nilsson et al., 2009; Rummer et al., 2014). The quantitative changes of DS genes *alas2* and *hpx* may suggest changes to the heme synthesis rate for oxygen supply and cell protection from excessive heme (Chiabrando et al., 2014). Although vasodilation and increase in vascular compliance can be induced by high temperature to augment the oxygen supply to the surrounding hepatic tissue (Thorne et al., 2020), hyperthermia can lead to chronic histopathologic changes in vascular network in liver such as hepatic necrosis, vascular congestion, and hemorrhage (Harper & Wolf, 2009). We found that *vascular endothelial growth factor a* (*vegfa*), which has a role in angiogenesis and hematopoiesis (Nieves et al., 2009), and *neurofilament heavy chain* (*nefh*), which has a function of axonogenesis (Sihag et al., 2007), were differentially spliced. The alternative splicing in these genes may be key to rebuilding damaged cells, as elevated temperatures can lead to a subsequent proliferation of liver cells in fish (Schultz et al., 1993) and enlarged fish livers (Bernal et al., 2018). These transcriptional changes may therefore indicate post‐damage recovery of the liver during the decline period of the heatwave.

Considering that genes involved with splicing were themselves differentially spliced in different heatwave periods, they may be regulated during environmental changes for maintaining gene functions and control of splicing patterns. The splicing factors and the splicing‐associated genes, such as *serine*/ *arginine rich splicing factor* 7 (*srsf7*), *KH*‐*type splicing regulatory protein* (*khsrp*), *small nuclear ribonucleoprotein polypeptide a’* (*snrpa1*), and *CDC Like Kinase 4* (*clk4*), were identified as DS genes. Splicing factors have also been identified as differentially spliced under thermal stress in catfishes (Tan et al., 2019) and rainbow trout (Sun et al., 2021), plants (Palusa et al., 2007), and mammals (Yamamoto et al., 2016). These genes directly participate in different aspects of the splicing process, including formation of the spliceosome complex, which may in turn control the splicing of other mRNAs under heat stress. A study on ﻿mice, for example, concluded that heat stress could lead to dephosphorylation of serine/arginine‐rich splicing factors (SRSF) which represses the normal splicing scheme, while CDC like kinase isoforms produced by alternative splicing showed the ability to recover the phosphorylation status of dephosphorylated SRSF rapidly after the heat stress (Ninomiya, Kataoka and Hagiwara, 2011). Hence, the alternative splicing mechanism may be flexibly controlled by the interaction of splicing proteins. This highly complex splicing regulation might have a significant contribution to plasticity of the fish proteome in response to thermal stress, and this response appears to be conserved across both plants and animals.

The collection of samples across different time points of the heatwave allowed us to evaluate DS during the onset, middle, and end of the acute warming. It is important to highlight that one of the main characteristics of this study is that our fish came from natural populations, which gives us direct evidence of how fishes respond to heatwaves in the wild. This though creates the limitation that other environmental variables could be confounding the effects of acute warming. For example, potential changes in food availability caused by the heatwave could not be directly investigated in this analysis. The acute warming of the ocean could also alter water chemistry and ambient biotic condition. Therefore, the effects observed in this study could represent the overall consequence of heatwave conditions in a marine fish, with the acute temperature increase as the main driver. Further, seasonal changes that are associated with the natural life cycle of *A*. *polyacanthus* remain unknown. Ideally, a “control year” without a heatwave would allow us to evaluate more in depth the processes driven by the heat exposure. However, the year after 2015/2016 was another year with a thermal anomaly and unfortunately such a collection of samples was not possible. Further investigations, also including different populations to validate the generality of the response (Veilleux et al., 2018), are needed to confirm that these observations are derived from temperature changes alone or whether other factors are also playing a role.

Heatwaves are projected to be longer and more intense in the future, which will compound the effects of increase average ocean temperatures (Masson‐Delmotte et al., 2021; Oliver et al., 2018). This is expected to have a considerable detrimental effect on marine poikilotherms, affecting the individual physiological response as well as community structure. Studying the wide variety of molecular responses related to warming can help us understand how wild populations of marine fishes will respond to a changing ocean. By using the 2016 marine heatwave as a case study, our results show that differential splicing was higher during the onset of the heatwave, for genes associated with the endoplasmic reticulum, Golgi apparatus, the immune responses, and splicing. Meanwhile, the transition from summer to winter was related with DS genes of heme‐related proteins and cellular growth, potentially a post‐damage recovery response after an intensive period of heat stress. The prolonged period of the heatwave revealed little changes in splicing patterns, suggesting that changes in splicing may be more relevant when individuals experience large changes in temperature over short time scales. Based on these results and previous studies, we suggest that changes of gene expression may be mostly responsible for maintaining the physiological and metabolic needs of *A*. *polyacanthus*, as this species probably has limited capacity of reversible thermal acclimation (Rodgers et al., 2018). Finally, the differential splicing patterns of splicing regulators themselves during heatwave periods may imply a fine splicing control network coupled with transcriptional changes, which allows the regulation of multiple cellular responses in the face of warming. The transcriptional changes and post‐transcriptional modification of the spliced gene products may assist *A*. *polyacanthus*, and possibly other coral reef fishes, to respond to the different stages of a marine heatwave. This study represents an example on how multiple molecular mechanisms acting in concert may help coral reef fish to acclimate the warming condition during marine heatwaves, as well as the potential processes related with compensation to long‐term ocean warming.

## CONFLICT OF INTEREST

The authors declare no competing interests.

## AUTHOR CONTRIBUTIONS

**Stanley Kin Nok Chan:** Conceptualization (lead); Formal analysis (lead); Investigation (lead); Methodology (lead); Project administration (lead); Software (lead); Validation (equal); Visualization (lead); Writing – original draft (lead); Writing – review & editing (equal). **Sneha Suresh:** Conceptualization (supporting); Methodology (supporting); Software (supporting); Supervision (supporting); Validation (equal); Writing – original draft (supporting); Writing – review & editing (supporting). **Philip Munday:** Conceptualization (supporting); Project administration (supporting); Validation (equal); Writing – review & editing (supporting). **Timothy Ravasi:** Conceptualization (supporting); Project administration (supporting); Validation (equal); Writing – review & editing (supporting). **Moisés A. Bernal:** Conceptualization (supporting); Project administration (supporting); Supervision (supporting); Validation (equal); Writing – original draft (supporting); Writing – review & editing (lead). **Celia Schunter:** Conceptualization (lead); Data curation (lead); Formal analysis (supporting); Investigation (supporting); Methodology (supporting); Project administration (lead); Resources (lead); Supervision (lead); Validation (equal); Visualization (supporting); Writing – original draft (lead); Writing – review & editing (lead).

### OPEN RESEARCH BADGES

This article has earned an Open Data Badge for making publicly available the digitally‐shareable data necessary to reproduce the reported results. The data is available at https://repository.kaust.edu.sa/handle/10754/662265?show=full.

## Supporting information

Supplementary Material

## Data Availability

RNA‐seq raw sequences and the *de novo* assembled transcriptome assemblies have been deposited in NCBI under BioProject PRJNA489934 and the sequences read archive (SRA) SRP160415.
